# Abiotic and Biotic Factors Associated with Tick Population Dynamics on a Mammalian Host: *Ixodes hexagonus* Infesting Otters, *Lutra lutra*

**DOI:** 10.1371/journal.pone.0047131

**Published:** 2012-10-05

**Authors:** Ellie Sherrard-Smith, Elizabeth Chadwick, Joanne Cable

**Affiliations:** Organisms and the Environment, Cardiff University, Cardiff, United Kingdom; Kansas State University, United States of America

## Abstract

The Eurasian otter, *Lutra lutra,* hosts several parasites with zoonotic potential. As this semiaquatic mammal has large ranges across terrestrial, freshwater and marine habitats, it has the capacity for wide dispersion of pathogens. Despite this, parasites of otters have received relatively little attention. Here, we examine their ectoparasite load and assess whether this is influenced by abiotic or biotic variables. Climatic phenomena such as the North Atlantic Oscillation (NAO) affect weather conditions in northern Europe. Consequently parasite distributions, particularly species with life stages exposed to the external environment, can be affected. We assessed the extent to which inter-annual variations in large-scale weather patterns (specifically the NAO and Central England (CE) temperatures) and host characteristics influenced tick prevalence and intensity. Ectoparasites consisted of a single species, the nidiculous tick *Ixodes hexagonus* (prevalence  = 24.3%; mean intensity  = 7.2; range  = 1–122; on n  = 820 otter hosts). The prevalence, but not intensity of infestation, was associated with high CE temperatures, while both prevalence and intensity were associated with positive phases of the NAO. Such associations indicate that *I. hexagonus* are most abundant when weather conditions are warmer and wetter. Ticks were more prevalent on juvenile than sub-adult or adult otters, which probably reflects the length of time the hosts spend in the holt where these ticks quest. High tick number was associated with poor host condition, so either poor condition hosts are more susceptible to ticks, or tick infestations negatively impact on host condition. Otters are clearly an important and common host for *I. hexagonus*, which has implications for vector-borne diseases. This work is the first to consider the impacts of long-term weather patterns on *I. hexagonus* and uses wild-animal cadavers to illustrate the importance of abiotic and biotic pressures impacting parasitic populations.

## Introduction

Current change in climate (the long-term average meteorological conditions of a region [1]) is associated with increases in temperature and precipitation, especially in Northern temperate zones [1]. This influences parasite distributions both directly [2], [3] and indirectly, for example via impacts on host range [4], [5]. Weather (short-term variation in meteorological conditions) can cause variations in parasite distributions whilst synchronously influencing host abundance [6] but will affect specific host-parasite interactions differently [4], [7]–[9]. Weather patterns are influenced by climatic phenomena such as the North Atlantic Oscillation (NAO). The NAO affects European climate such that, when in positive phases, northern Europe experiences warmer and wetter conditions [10], [11]. Identifying associations between climate and the distribution of vectors over time (e.g. [12], [13]) is an essential pre-requisite to understanding public and wildlife health risks resulting from vector-borne infection.

Ixodid ticks are vectors for a range of pathogens causing diseases including Lyme disease, Boutonneuse fever, Q fever, tick-borne fever and tick-borne encephalitis [14]. *Ixodes hexagonus* is an efficient vector of *Borrelia burgdorferi,* the causative agent of Lyme disease [15] but in the UK, *I. ricinus* has received most attention because of its ubiquitous nature and association with transmission of pathogens to humans and livestock [14]. The distribution of *I. ricinus* is influenced by weather [16], [17] and the presence of suitable hosts and habitat [18]. Temperature increases are associated with increasing population density and geographic range of *I. ricinus,* a European tick [17], [19], and other tick species such as the North American species *I. scapularis.* [20]. The majority of ixodid ticks require >80% relative humidity for survival off the host [21]–[23] and as such, positive phases of the NAO may benefit ixodid ticks by creating suitably humid weather. Landscape, habitat use and local weather conditions have been associated with tick distributions previously [16], [17], [19], [20], [24], [25]. The impact of such environmental variables on host-parasite interactions is, however, highly variable [26]. Mustelids have been associated with the nidiculous (burrow or nest dwelling) tick *I. hexagonus* [24] but the relationship between *I. hexagonus* and weather conditions has not been examined previously.

The Eurasian otter, *Lutra lutra*, is a top predator in the UK and a sentinel of freshwater health [27]. Otters are wide ranging opportunistic predators that feed in terrestrial, freshwater and marine habitats [28]. They are therefore potentially exposed to a wide diversity of pathogens and a great deal can be learned about the distribution of parasites in UK ecosystems by screening such a generalist host. Here, we identified the tick species that use otters as a host. Next, we investigated how weather patterns and host characteristics are associated with tick infestations of otters in England and Wales. Specifically, we hypothesised that tick occurrence (prevalence and intensity) would be positively correlated with temporal variation in: i) the NAO (associated with warmer and wetter weather in the UK), and; ii) higher Central England (CE) temperatures (a long-term record of temperature in central England, see Materials and Methods). Based on these findings we hypothesised that spatial variation in tick counts among meteorologically distinct regions of the UK would correlate positively with rainfall and temperature.

## Results

### Tick species

**Figure 1:**
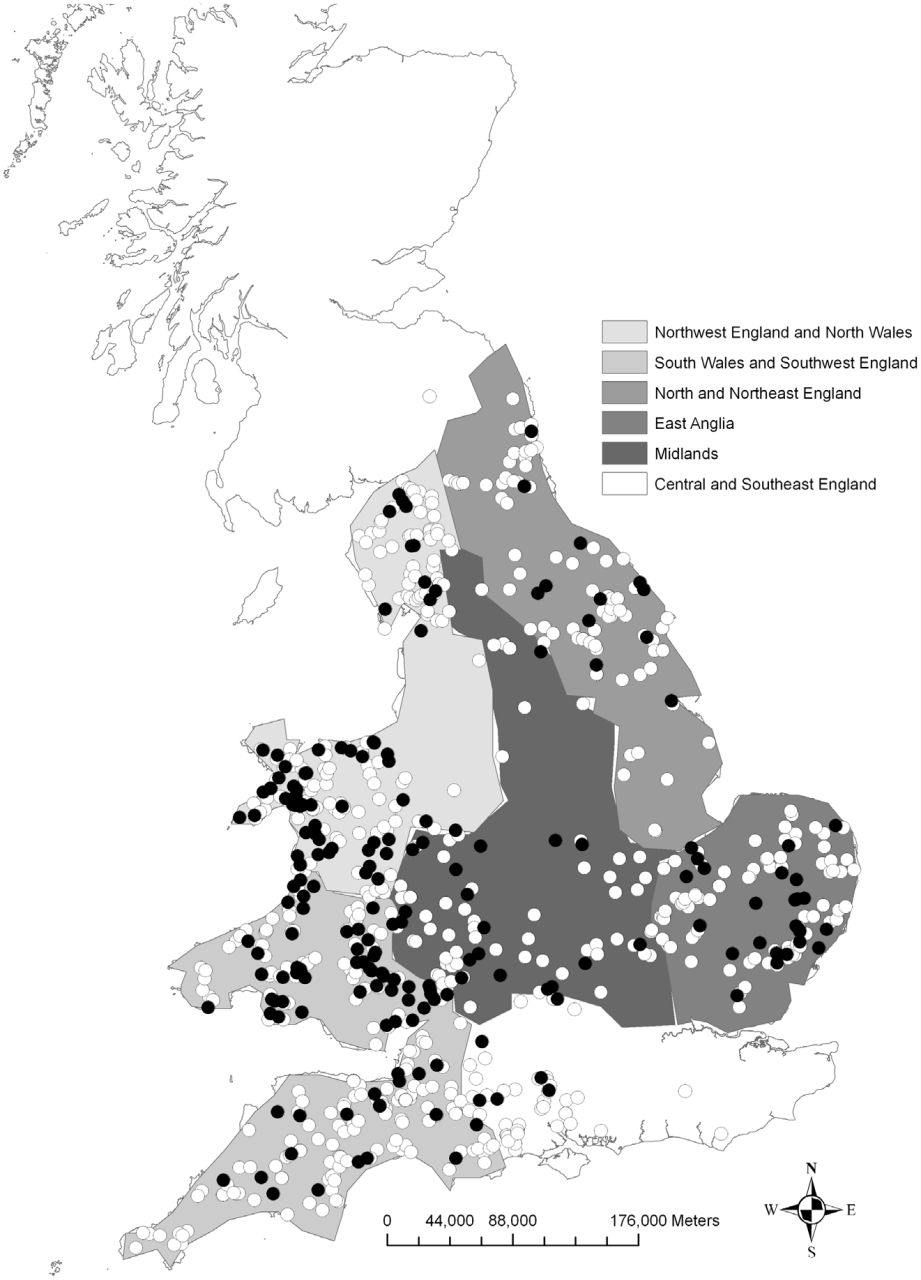
Tick distributions across the UK. Distribution of *Ixodes hexagonus* infested (dark circles) and uninfested (clear circles) otters in England and Wales. Meteorologically distinct regions (East and Northeast England, East Anglia, Southeast England and Central South, Northwest England and North Wales, South Wales and Southwest England, and Midlands) defined by the Meteorological Office UK Climate Impacts Programme (data available online).

Tick (*Ixodes hexagonus*) prevalence on Eurasian otters, between 2004 and 2010, was 24.3% (199 out of 820) (Figure 1). On some hosts, all post-hatch tick life stages (larva, nymph and adults) were recovered (18 cases), but almost 40% of hosts had only one life stage present at collection (Larvae  = 13 cases, Nymph  = 40 cases, Adult  = 26 cases) (Table 1). Infested otters were widespread across England and Wales (Figure 1) with no evidence of clustering of infestation within the otter distribution (Ripley's K analysis at the 95% confidence level using radii ranging from 1 km-130 km).

**Table 1:** Summary of *Ixodes hexagonus* on otters.

Parasite stage	Prevalence (%)	Count (/820 hosts)	Mean Intensity (95% CI)	Range
Any stage	24.3	199	7.2 (5.5–9.2)	1–122
Larva	9.3	76	7.7 (4.7–11.7)	1–112
Nymph	15.1	124	4.0 (2.9–5.2)	1–44
Adult	11.8	97	2.7 (2.1–3.5)	1–26

*Ixodes hexagonus* infestations of *Lutra lutra* in England and Wales between 2004 and 2009 (n = 820); showing prevalence, parasite count, mean intensity with upper and lower 95% bootstrap confidence interval (10000 iterations), and maximum intensity for each tick life stage.

### Abiotic factors

**Figure 2:**
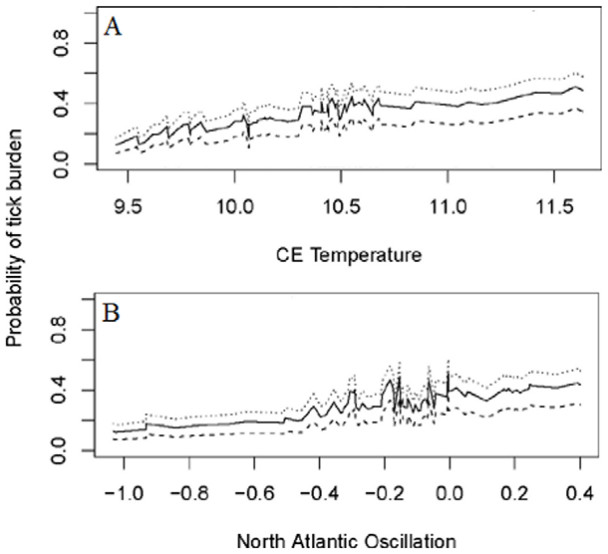
Abiotic impact on tick prevalence. Probability plot for a model of the association between tick prevalence and the explanatory variables A) Central England Temperature for the 12 month period preceding host death, B) North Atlantic Oscillation for the 12 month period preceding death for each host age class: Dotted line  =  juvenile hosts; Solid line  =  Adult hosts; Dashed line  =  Sub-adult hosts.

**Figure 3:**
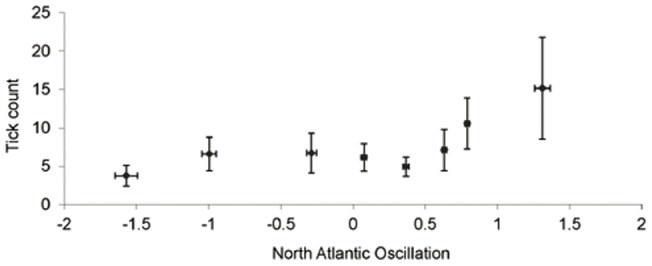
The North Atlantic Oscillation impacts tick counts on otters. Relationship of tick count to mean North Atlantic Oscillation at month of host death. Standard error bars shown.

Tick prevalence on otters was associated with higher Central England (CE) temperatures for the 12 month period preceding host death (GLM: t  = 2.594, df = 569, p<0.01), and more positive phases of the North Atlantic Oscillation (NAO) over the 12 month period preceding host death (GLM: t = 2.099, df = 569, p<0.05) (Figure 2). Tick intensity was not significantly associated with CE temperatures over any period preceding host death (GAM: t = 1.445, df = 155, p = 0.15). Tick intensity was, however, positively associated with the NAO at month of host death (GAM: t = 2.670, df = 155, p<0.05) (Figure 3).

**Figure 4:**
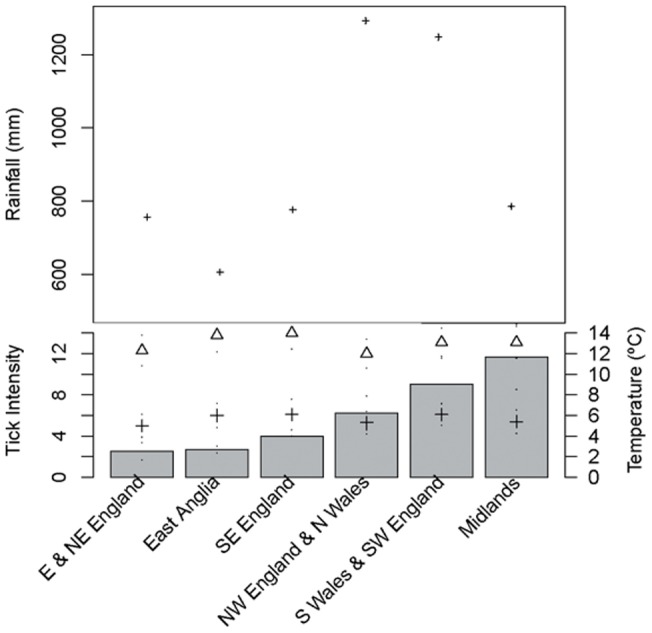
Abiotic impact on tick intensity. Mean tick intensity (grey bars) in each meteorologically distinct region (East and Northeast England, East Anglia, Southeast England and Central South, Northwest England and North Wales, South Wales and Southwest England, and Midlands) and corresponding 30 year average (1971–2000) summed mean rainfall (mm) (upper Y-axis), maximum (triangle) and minimum (cross) 30 year (1971–2000) average temperature (°C) for each region (lower Y-axis). Standard error marks for rainfall, maximum and minimum temperature correspond to variability in monthly averages.

The South Wales and Southwest England region (Figure 1) had significantly higher tick counts on otters than all other regions, while East and Northeast England had significantly lower tick counts than all other regions; these two regions contributed most strongly to the statistically significant difference in counts between regions (χ^2^  = 302.169, df = 5, p<0.001). Mean intensities for each region did not, however, correlate with maximum or minimum temperature, or mean rainfall for the long-term yearly average (1971–2000) regional data (Correlations, all p>0.1) (Figure 4). There were no seasonal associations between larval, nymph or adult stage ticks on otters (GLM, p>0.1).

### Biotic factors

**Figure 5:**
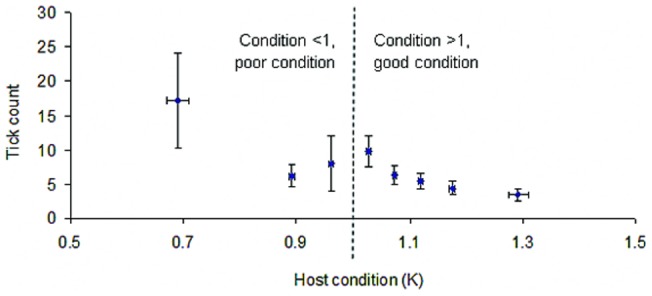
Biotic impact on tick count. Relationship of tick intensity to host condition (K). Standard error bars shown.

More juvenile otters were infested than older age-classes (GLM: p>0.01; Figure 2). The mean host condition ‘K’ for the sampled population was 1.0286. Tick intensity was inversely related to otter condition so that as otter condition increased, tick intensity decreased (GAM: t = 3.137, df = 155, p>0.01) (Figure 5).

## Discussion

*Ixodes hexagonus* is the only tick species reported from the Eurasian otter ([29], current study). *Ixodes hexagonus* can complete its life cycle on the European hedgehog [24], fox [30] and American Mink [31]. As all three post-hatch tick life stages were found on the otter in the current study, it appears that *I. hexagonus* can also potentially complete its life cycle on this mammal. The prevalence of *I. hexagonus* on otters (24.3%) is lower than that reported on European hedgehogs, which are the preferred host for this tick [24], [32] (53.3% prevalence on hedgehogs from Western Europe, [32]). *I. hexagonus* is encountered by domestic dogs and cats in urban areas [33] illustrating the close proximity of this particular tick to human populations. The prevalence of *I. hexagonus* on otters is, however, high in comparison to its prevalence on the domestic dog in the UK (5.6%, n = 3534 [34]). Further, the mean intensity (the total number of parasites of a particular species found in a sample divided by the number of infested hosts [35]) of ticks is higher on otters (7.2 per host) than on hedgehogs (3.8 [36]) despite examination of cadavers in the current study and live hosts in the hedgehog study. This suggests that otters are a noteworthy host for *I. hexagonus*. The association between otters and *I. hexagonus* populations may be important for pathogen transmission, particularly if otters act as either reservoir or amplifier hosts, or reduce pathogen abundance through the dilution effect [37]. Further, otters have large home ranges [28], [38] indicating that this host has the potential to transfer ticks between habitat islands. The nocturnal and aquatic nature of otters may deter other tick species from utilising such a resource, explaining the absence of diversity in tick species.

Positive phases of the North Atlantic Oscillation (NAO) were associated with increased prevalence and intensity in tick populations on otters. Strong positive phases of the NAO are linked with above average temperature and precipitation across northern Europe. Together with the elevated humidity produced, such weather conditions may lead to increased abundance of *I. hexagonus*, as reported for *I. ricinus* and *I. scapularis* [17], [19], [20]. This may be related to the weather conditions causing changes in the behaviour of either the parasite or the host thereby altering infestation rates (see [39]). No previous literature was found relating NAO to *I. hexagonus*. In *I. ricinus* however, the NAO did not correlate with intensity of tick infestation but negative winter NAO phases (associated with warmer and wetter winters) corresponded to increased Borreliae infections [40]. Further investigation into the underlying pathogenic infections of otters would be useful to examine whether this association holds for *I. hexagonus.*

Both NAO and CE temperatures are indices that can be used to describe temporal variation in weather; they do not provide spatially explicit weather data within the UK. The significant relationships found in the current study therefore describe an association between otter ticks (prevalence and intensity) and temporal variation in weather. We tested subsequently whether warmer and wetter regions were associated with higher tick infestations of otters, and found significantly more ticks in South Wales and Southwest England – a region associated with higher rainfall [41]. Overall, however, at the regional scale we identified no significant correlations between the mean intensity of ticks and either temperature or rainfall. This may be because at the regional scale temperature and rainfall are negatively correlated, so a more detailed analysis of local weather is necessary to clarify their interaction. Other factors such as the distribution of non-otter hosts, and variation in habitat type, may also heavily influence spatial variation in tick abundance. In preliminary investigations we explored the impact of local weather, alternative hosts, and habitat on *I. hexagonus* distribution, but subsequently removed these from our analyses because: i) Restricted availability of data meant that inclusion of both spatial and temporal variation in weather reduced the size of the dataset considerably, rendering conclusions less robust; ii) Information on the reported distribution of alternative hosts (hedgehog and fox) and of *I. hexagonus* were obtained from the National Biodiversity Network (NBN). Hedgehogs and foxes are both widespread and abundant in the UK and therefore availability of alternative hosts seems unlikely to limit *I. hexagonus* distribution at the regional scale. Further, *I. hexagonus* records from the NBN are concentrated in the London area of South East England, but because this database relies heavily on records submitted by members of the public this is likely to represent bias due to distribution of the human population. The NBN records map presence only (and not absence on screened hosts), so it was not possible to test for clustering within the host distribution as we did for *I. hexagonus* on otters. Comparisons were therefore uninformative; iii) Data on land use (arable, broadleaf and coniferous woodland, improved and semi-natural grassland, and upland habitat) were obtained from the Countryside Information System (CIS version 8, available online). ArcMap GIS (version 9.2) was used to interrogate these data and to assign percentage cover of each land-use within a 20 km radius of each otter. Significant negative associations were revealed (between tick prevalence and arable land, improved grassland, and conifer woodland), but interpretation is questionable because of the heterogenous and patchy nature of habitat data, the relatively large areas examined which may not accurately reflect the nature of real otter ranges (these tend to be linear along water courses, and vary considerably in length from a few to 40km [28]), and the difficulty in defining where, within this unknown range, an otter may have become infested.

Tick prevalence, but not intensity on otters, was associated with CE temperature. As far as we are aware, there are no previous records of temperature effects on *I. hexagonus* and the only long-term study on population dynamics of *I. hexagonus* indicates little seasonal variation and low-level abundance (on hedgehogs [36]). In general, however; temperature has a key role in driving tick development rates [17], [19] and so affects population dynamics [16], [17]. Additionally, temperature tends to be associated with length of diapause, larval activity and adult interactions [42]. Particularly strong associations are found between *I. ricinus* and temperature [17]. Stochastic temperature variations across the year are predicted to alter population dynamics of *I. ricinus* with subsequent impacts on the transmission of vector borne diseases [17]. The contrasting impact of temperatures on *I. hexagonus* and *I. ricinus* may be attributable to the ecological differences between the two species. The most important of these is likely to be habitat choice. *I. hexagonus* is nest dwelling, and so to some extent insulated from changes in ambient temperatures. In contrast, *I. ricinus* uses open areas for questing [14], so is likely to be exposed to wider fluctuations in air temperature.

Juvenile otters were more frequently infested with *I. hexagonus* than adult hosts. Host age, in general, influences the intensity of infestations, but can also affect parasite-induced mortality, and the distribution of the parasite among host individuals [43]. Several hypotheses [43], [44] predict that juveniles will carry heavier infestations than older hosts, either because: i) adult hosts develop immunity and/or behavioural adaptations to avoid or remove parasites; and/or ii) heavily infested juveniles die before adulthood (selection hypothesis [44]) although this is very unlikely as a direct cause of death. Grooming is a learned activity in otters [28] and may contribute to lower tick numbers on older otters [28]. Additionally, young otters spend the majority of their time in holts, the resting place of otters [28], so are disproportionately exposed to such parasites. Effects may be underestimated here, however, because road killed samples tend to reflect the healthier section of the population [45].

Finally, we found a relationship between host condition and tick intensity such that a better host condition is associated with decreased intensity. This is not a reflection of the elevated infestations on juvenile hosts because the host condition index used here [46] controls for size and therefore age, in addition to sexual dimorphism. This positive relationship could imply that otters in better condition are more efficient at grooming and thereby rid themselves of ticks, or that ticks have a negative impact on otter condition.

We acknowledge that data from road-killed hosts are likely to underestimate tick counts and recognise that road-kill samples are a stochastic sub-sample of a population and may lead to bias in terms of the proportion of the host population examined. For protected species, however, road-kill samples remain the only way to obtain large sample sizes for analysis. The absence of tick species other than *I. hexagonus,* in concordance with the only other report of otter tick infestations [29], could reflect differences in emigration patterns when abandoning a dead host, while tick emigration rates from dead hosts may interact with local microclimate. Our analysis of recently killed versus decomposed otters (see Materials and Methods), however, reveals no significant difference in infestation levels or species diversity, suggesting that observed associations are robust. Such data can therefore successfully illustrate associations between inter-annual variations in weather patterns, host characteristics and *I. hexagonus* populations.

To our knowledge this work is the first to consider the impacts of weather on *I. hexagonus,* and reveals that inter-annual variations in large-scale weather patterns, together with host characteristics, combine to affect the distribution, prevalence and intensity of *I. hexagonus* on Eurasian otters. Associations were identified between positive NAO phases, CE temperatures and tick prevalence, suggesting that the predicted change in climate in northern temperate zones may cause an increase in *I. hexagonus* populations. Although the associations highlighted here may not necessarily parallel what is observed on other hosts for this tick, *I. hexagonus* is common on domestic cats and dogs [33] and we suggest that tick research should, perhaps, target species other than *I. ricinus* in the future. This study illustrates how surveys of wild-animal cadavers can be hugely informative about parasitic populations.

## Materials and Methods

### The host

The Cardiff University Otter Project receives dead otters, *Lutra lutra*, from across England and Wales. Most (86% of the current study) have been killed by road traffic and are stored subsequently at −20°C. The location (British National Grid Reference) and date of death (month and year), sex, age-class (juvenile (n = 25), sub-adult (238) and adult (312) and size (weight (kg) and length (m)) were recorded for each otter collected between 2004 and 2010. A condition index K was calculated controlling for the dimorphism of otter sexes, following [46]. Such that:
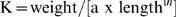
where a  = 5.02 and *n* = 2.33 for females, and a  = 5.87 and *n* = 2.39 for males [46]. Seasons were defined as winter: December-February, spring: March-May, summer: June-August, and autumn: September-November. Very decomposed otters were excluded from the analysis. Remaining otters included in model (and excluding those with missing data; n = 575) were distributed across seasons and years as follows: spring  = 137; summer  = 83; autumn  = 179; and winter  = 176; 2004 = 12; 2005 = 48; 2006 = 70; 2007 = 122; 2008 = 146; 2009 = 116; and 2010 = 61.

### Parasite identification

Ticks were removed (via pelt searching and fur combing) and stored in 90% molecular grade ethanol prior to immersion in 0.1% saline solution for microscopic examination (x30 magnification) using a Nikon dissecting microscope with fibre optic illumination, and identified to species using morphological features [14], [47]. *Ixodes hexagonus* was the only tick species present and species identification of 15 specimens (5 adults, 5 nymph and 5 larvae) was confirmed by the Natural History Museum. Occasionally, damage or desiccation prevented morphological identification so for these specimens we sought confirmation using mitochondrial DNA cytochrome oxidase sub-unit 1 (COX1) analysis as follows.

DNA was extracted from 13 specimens, three adults, three nymphs and seven larvae, from four geographically separate hosts. Ethanol was evaporated fully from each sample. Extractions were conducted using a QIAGEN kit as per the manufacturer's protocol (QIAGEN DNeasy Blood and Tissue Handbook 2006) with the additional step of manually crushing each tick body with a sterile pipette tip at the start of the process. PCR followed standard procedures (QIAGEN DNeasy Blood and Tissue Handbook 2006). Novel primers (IHEXCO1F: 5′- TCATAAAGACATTGGGACT-3′, IHEXCO1R: 5′- TGGTAAAGAATGGGGTCT-3′) were designed by alignment of COX1 mtDNA from 8 reference tick species (GenBank: *Dermacentor reticulatus* AF132829, *Haemaphysalis punctata* FN394339.1, *Hyalomma aegyptium* AF132821, *Ixodes uriae* NC006078, *I. hexagonus* AF081828.1, *I. lividus* GU124743, *I. ricinus* FN394342 and *Rhipicephalus sanguineus* NC002074). These primers are specific to *I. hexagonus.* The PCR reaction conditions were carried out in a 50 µl final volume, with 10x PCR buffer II (Applied Biosystems, UK), 50 mM MgCl (Applied Biosystems, UK), 2.5 mM of each dNTP, 10pmol/µ l of each primer, 0.5 U Taq DNA polymerase (Invitrogen) for each 10 µl DNA template. PCR conditions (GenAmp PCR System 9700, Applied Biosystems, UK) were: 95°C for 5 min, followed by 35 cycles of 94°C for 30 sec, 53°C for 1 min and 72°C for 1 min, with a final extension of 72°C for 10 min. PCR products produced identical sized bands for all tick samples on a 1.5% agarose gel. Four larvae, one nymph and one adult were sequenced (QIAGEN, Genomic Services, Germany) using both forward and reverse primers. All 592 bp sequences from the current study were identical and showed 99% similarity to the corresponding region of GenBank *I. hexagonus* AF081828.1. This reference sequence, AF081828.1, was obtained from laboratory maintained ticks over ten years ago [48], perhaps explaining the 3 bp discrepancy (at position 130 transition T to C, and at positions 172 and 188 transversions A to C). The next closest sequence match was 82% with *Ixodes asanumai* Kitaoka 1973 (GenBank: AB231674.1).

### Data preparation

Temporal variation in weather was quantified using mean monthly temperatures (°C) for Central England (CE temperature) [49] and North Atlantic Oscillation (NAO) phases (http://www.cpc.ncep.noaa.gov/products/precip/CWlink/pna/nao.shtml, data provided by the Climate Prediction Centre of the U.S. National Oceanographic and Atmospheric Administration website). The mean of each was calculated for: i) the month of host death; ii) the sixth month period preceding host death; and iii) the year preceding host death, for each otter. These time periods were selected based on literature indicating that populations may be influenced by conditions during the previous season or year [16], [50].

Spatial variation in climate was quantified using long-term averages, which are a useful tool to describe the state of the climate in a particular region [41]. Long-term yearly average (1971–2000) temperature (maximum and minimum, °C) and rainfall (mm) measures for meteorologically distinct regions of England and Wales were collated [41]. These regions are defined as East and Northeast England, East Anglia, Southeast England and Central South, Northwest England and North Wales, South Wales and Southwest England, and Midlands (Figure 1) and are used by the Meteorological Office UK Climate Impacts Programme (UKCIP) to summarise weather patterns in the UK (http://www.metoffice.gov.uk/climate/uk/averages/19712000/). To determine the abiotic conditions for each sampled carcass, otters were assigned the regional average for climate data depending on their geographic location at time of death. Associations between these measures of climate and tick prevalence (the number of hosts infested with specific parasitic species, in the current study ticks, divided by the total number of hosts examined [35]), intensity (the number of individuals of a particular parasite species on a single infested host [35]) and tick count (the total number of ticks within a population) were examined from otters found between 2004 and 2010.

### Data analysis

Ectoparasites are thought to abandon dead hosts [51]. We tested initially, therefore, whether there was a difference in tick abundance between fresh (collected within 24 h of death, n = 610) and not fresh (otters characterised as slightly or moderately decomposed, n = 210) otters. We found no significant difference in tick presence/absence (χ^2^ = 0.515, n = 820, df = 1, *P* = 0.473) or median intensity (Kruskal-Wallis H = 0.35, n = 195, df = 1, *P* = 0.556) and subsequently pooled all data for further analyses.

The NAO and CE temperatures, for each time period examined, and host factors (sex, age, condition, season and year of death) were combined in a general linear model fitted to the tick presence/absence data with a binomial error distribution (n = 575 hosts, individuals were omitted where data was missing). A generalised additive model incorporating these explanatory variables was fitted to the tick intensity data (tick counts per otter excluding zero counts) with negative binomial error distribution. Relationships between explanatory variables and tick intensity were non-linear. A generalised additive model (GAM) was applied therefore with splines fitted appropriately. Final models were selected using Akaike Information Criterion (AIC).

Tick counts were compared between the meteorologically distinct regions of England and Wales (described above) by calculating regional mean tick intensities and testing for a correlation with the long-term yearly average maximum and minimum temperature (°C) and total rainfall (mm) in each region.

The spatial distribution of infested otter carcasses (n = 199) was examined to look for clustering within the host distribution (n = 820) by calculating a modified Ripley's K statistic, K[i.](r), using Ripley's isotropic edge correction [52] with a simplified border of England and Wales as a boundary (for further details of methodology [53]). All statistical analyses were conducted using R version 2.12 [54].
